# The Retina in Cases of Albuminuria

**Published:** 1908-06-06

**Authors:** Leslie Paton

**Affiliations:** Assistant Ophthalmic Surgeon and Lecturer on Ophthalmology, St. Mary's Hospital; Assistant Ophthalmic Surgeon, National Hospital for the Paralysed and Epileptic.


### THE RETINA IN CASES OF ALBUMINURIA.

By LESLIE PATON, M.B., F.R.C.S.

Assistant Ophthalmic Surgeon and Lecturer on Ophthalmology, St. Mary's Hospital; Assistant Ophthalmic Surgeon, National Hospital for the Paralysed and Epileptic.

(Abstract of a lecture delivered at the Medical Graduates' College and Polyclinic.)

It is hardly necessary for me to point out the value and the importance of a thorough ophthalmoscopic examination of the retina in all cases of albuminuria. The value of such an examination is not so much diagnostic as prognostic. There are, of course, cases in which the appearance of the retina, as seen by ophthalmoscopic examination, is of diagnostic importance; but, practically, the great value of such examination lies in the fact that it enables us to speak more decidedly on the question of prognosis.

It has been known for a long time that certain cases of albuminuria are associated with retinal changes. As early as 1812 Wells drew attention to certain cases in which blindness was associated with dropsy, and Bright in 1827 described the association of defective vision and blindness with kidney changes. But it was not till 1851 that Turck described definite macroscopic and microscopic changes in the eye in cases of albuminuria, and in 1859 Liebreich, in his atlas, gave the first ophthalmoscopic picture of the retinal changes in similar cases.

### Types of Nephritis.

Before proceeding to detail the lesions which are met with in the retina, it is, perhaps, advisable to recapitulate the main types of nephritis which one meets with. It is difficult directly to associate any one definite nephritic change with any one definite retinal change, and it is therefore necessary to go into some detail regarding the various forms of nephritis.

We may put out altogether the so-called *Cyclical albuminuria*, which has no bearing on this subject of retinal changes, although it is true that in a few isolated cases some observers have put on record slight changes which have been found on examination. Also we may put out both *Suppurative nephritis* and *Amyloid disease*. Bull in America has reported two cases of amyloid disease associated with retinal changes, but these are most exceptional, and it is probable that in both the condition was one of early amyloid changes in the retina itself. I will restrict my remarks to the more common types of kidney disease.

The simplest form of nephritis is that associated with acute fevers, such as scarlet and diphtheria, and which is sometimes met with during pregnancy. This form affects the tubules chiefly, and leaves little or no permanent damage. There is cloudy swelling of the epithelium of the tubules, with a varying amount of albumen in the urine, a small quantity of blood and a few tube casts. This type is practically never associated with definite retinal changes.

The next type is *Acute diffuse nephritis*, which follows acute specific fevers, prolonged exposure to cold, especially when the patient is an alcoholic, and which may be due to alcohol itself or to the action of one or other metabolic toxin. The kidneys are enlarged, and there is definite increase in size of the glomeruli and tubules. The quantity of excreted urine is diminished, the specific gravity of the urine varies inversely with the quantity excreted, the amount of albumen is large, and there are many casts. The amount of urea is diminished, while the pre-ureic bodies, such as xanthin, are proportionately increased, and the urine is acid in reaction. In this form both the stroma and the parenchymatous tissue of the kidney are affected.

There is a form of *Acute interstitial nephritis* which is characterised by a great increase in the number of plasma cells in the stroma and interstitial tissue. It is a form which occurs more in children than in adults, and there are no special eye changes.

Another type is *Chronic diffuse nephritis*. This may follow the acute variety. The parenchymatous tissue degenerates and the interstitial tissue proliferates in order to replace the higher type of tissue which has been destroyed. Yet this type may arise without any previous acute attack. Like the acute variety, it is chiefly met with in men, and produces the common large white kidney. The amount of urine is diminished and its specific gravity decreased; it holds much albumen, the urea, chlorides and phosphates are diminished, and many hyaline, granular, and fatty tube casts are found.

This is a form of nephritis which most usually affects men in early adult life, from twenty to forty years of age, and it may pass into *Chronic interstitial nephritis of a secondary type*, in which the interstitial changes are much more prominent. In this the kidneys will vary in size according to the stage of the disease until you get the small white kidney, in which the contraction is chiefly due to the increase in the amount of fibrous tissue. This kidney is the sclerosed or cirrhotic kidney. The capsule is adherent and peels off with difficulty: the glomeruli and tubules have undergone atrophy and hyaline degeneration in part, and in part are dilated, and the interstitial tissue has increased at the expense of the parenchymatous. In this condition the urine may be normal or it may be diminished: there is usually much albumen, and hyaline, epithelial, and fatty casts may be found.

The small granular kidney—*Chronic interstitial nephritis of a primary type*—may be met with in gouty conditions, in alcoholic poisoning and syphilis, and, to a certain extent, in lead-poisoning. Usually the patients are above forty. The kidney is small and shows an overgrowth of the connective tissue, the capsule is adherent, and the glomeruli show hyaline degeneration and may be markedly hypertrophied. The urine is greatly increased in quantity, as much as seven to ten pints being passed per day; the specific gravity is low (1005-1010), albumen, especially in morning urine, may be absent, or at least in very small amount, and the urea is diminished.

Associated to this type is the arterio-sclerotic kidney, which is practically the granular kidney with arterio-sclerosis. The kidney changes in this case start round the smaller arterioles, which microscopically show definite thickening of the inner coats. An examination of the retina may show very early changes in arterio-sclerosis—long, in fact, before any change is apparent in the urine to point to the fact that the patient is suffering from any kidney trouble.

### Retinal Changes in Albuminuria.

If you examine the eye in such an early case of arterio-sclerosis you will find slight, but very characteristic appearances. Later these appearances are more striking.

The first thing that you will notice is that the arterial coats show very definite thickening. They are running a straighter course than normal, their coats have a whiter colour, and the central streak alongside each shows up much more brightly. These are the appearances of the larger arteries. The smaller arteries, on the contrary, may seem to be more tortuous. Where an artery crosses a vein these changes are very striking. Normally the proportion of the artery to the vein is as 2 to 3, but in these cases it may be as low as 1 to 3. Normally, again, the arterial wall is translucent, so that the vein can be seen beneath it; now, however, it is quite opaque.

The vein is dimpled, distally dilated, and definitely compressed by the artery. There may be white lines running along the artery, but they are not to be relied upon as diagnostic of arterial thickening, as they are sometimes observed in a perfectly healthy disc. The thickening of the artery shortens and pulls out the curves, and the vessels therefore appear straight and white, so as to warrant the term " silver-wire arteries " applied to them by Marcus Gunn.

This is the typical appearance seen in arterio-sclerosis and associated with the arterio-sclerotic kidney. At times it may be accompanied by a slight degree of œdema. A patient with this kind of change is much more susceptible to the action of toxins, and is far more likely to get tobacco amblyopia than the man whose arteries are normal.

A somewhat similar appearance is seen in the patient with the small granular kidney, where the blood-pressure is high and the right ventricle is hypertrophied. In this case there is, perhaps, slightly more œdema, which chiefly affects the macular region, along with tiny hæmorrhages and ecchymoses.

A succession of such œdemas gives rise to the next form—the typical star-shaped albuminuric retinitis. In this there is a series of white patches round the fovea, together with œdema and hæmorrhages. The origin of this star-shaped appearance is probably due to the œdema. When the retina is œdematous it tends to fall into folds which radiate from the two fixed points, the disc and the macula, and gradually the œdema subsides into the hollows of these folds, giving rise to the characteristic star-shaped appearance. This is the type of retinal change which Gowers calls his first or degenerative type, the second being the type which is more specially associated with retinal hæmorrhages. I am inclined to class these two types together, as the difference between them is one of degree merely.

The third type, however, is a distinct and further advance. In this the œdema is much more severe, and the nerve head is involved, so that the condition is really one of definite neuro-retinitis. This is the type which is likely to be found in patients who have a large white kidney which is contracting down to the small form.

The edges of the optic disc are seen to be blurred; the arteries are slightly concealed and drop over the edge of the swelling; the veins are dilated and tortuous, and there are patches of flame-shaped hæmorrhage, more marked between the macula and the disc, with equally definite and sometimes much more pronounced patches of whitish-grey exudate —areas of lymphorrhage, in fact. This type is sometimes replaced by a definite albuminuric neuritis which is exactly similar to that seen in cases of cerebral tumour. The swelling here is very marked, and may be as much as five dioptres, or more, and it is practically impossible, from the retinal examination alone, to tell whether you are dealing with a case of cerebral tumour or with one of albuminuria.

These last stages, neuro-retinitis and neuritis, are apt to occur in young adults. Often the first sign of kidney-trouble is a dimness of vision, which leads the patient to seek advice, and then when the eyes are examined the condition found immediately raises the question of the integrity of the kidneys.

The serious point in such cases is the question of prognosis. Many cases showing these latter forms of change die within six months, and probably very few live more than a year. One must, however, except from this statement the retinitis associated with the albuminuria of pregnancy, to which this very grave prognosis does not apply. The importance of recognising this type of retinal degenerative change is therefore very great. The two first types of retinal change are usually met with in older patients above 40, and the outlook, though grave, is not so black as in the other cases. The risk in such cases is of some cerebral vascular lesion.

### Treatment.

The diagnosis of such conditions is important from the point of view of treatment. If cases of the first two types are seen early enough, much may be done for them. Where the heart is acting well and there is no risk of thrombotic changes, a reducing diet and regimen are indicated. Usually the average case is of this nature, and the pulse-tension can be lowered by small doses of calomel and potassium iodide, with rest and the avoidance of an over-high nitrogenous diet. But in cases where the heart is not acting well, there is danger of thrombosis, and reducing treatment may often do great harm.

